# Temporal Stability of Repeated Self-Reported vs Device-Measured Behavior: Observational Study Using Ecological Momentary Assessment

**DOI:** 10.2196/99953

**Published:** 2026-10-23

**Authors:** Meynard John L Toledo, Stefan Schneider, Joshua Smyth, Doerte Junghaenel, Arthur Stone

**Affiliations:** 1 Dornsife Center for Self-Report Science & Center for Economic and Social Research, University of Southern California, 645 W Exposition Blvd, Los Angeles, CA, 90089, United States, 1 7063632866; 2 Department of Psychology, University of Southern California, Los Angeles, CA, United States; 3 Leonard Davis School of Gerontology, University of Southern California, Los Angeles, CA, United States; 4 Department of Psychology, The Ohio State University, Columbus, OH, United States

**Keywords:** accelerometry, ecological momentary assessment, self-report reactivity, physical activity, sedentary behavior

## Abstract

**Background:**

A central concern in ecological momentary assessment (EMA) research is whether repeated self-report alters the reporting process itself. In most EMA domains, longitudinal stability of reporting is assumed but rarely tested.

**Objective:**

This study leveraged concurrent accelerometry as a temporal anchor to evaluate (1) whether self-reported physical activity (PA) and sedentary behavior (SB) exhibited temporal shifts across a 7-day monitoring period and (2) whether this temporal shift differed across near-momentary (5-min reporting period) and coverage-based (120-min recall period) EMA protocols.

**Methods:**

We conducted a secondary analysis of 315 US adults recruited from the Understanding America Study panel between January and July 2023 for the current study. Participants were monitored in their natural environments for 7 days via 2 measurement methods: continuous thigh-worn activPAL4 accelerometry and 5 daily smartphone EMA prompts assessing PA and SB. Reporting reactivity was defined as divergence in the temporal trajectories of EMA-reported behavior relative to accelerometry. Multilevel models with random intercepts and slopes tested whether linear and quadratic study day effects differed across the 2 methods (method × study day interactions). Protocol differences were examined using multivariable method × protocol × study day interactions, comparing coverage-based and near-momentary EMA designs.

**Results:**

In the coverage-based protocol, EMA-reported PA and SB exhibited temporal trajectories that diverged significantly from concurrent accelerometry (SB linear divergence: *b*=1.40, 95% CI 0.20-2.61, *P*=.02; PA linear divergence: *b*=−1.91, 95% CI −2.93 to −0.90, *P*<.001; and PA quadratic divergence: *b*=0.28, 95% CI 0.12-0.44, *P*<.001). No evidence of such divergence was found in the near-momentary protocol. The divergence in reporting trajectories differed significantly between the 2 protocols (SB linear 3-way interaction: *b*=−2.58, *P*=.02; PA linear 3-way interaction: *b*=1.86, *P*=.04).

**Conclusions:**

Reporting reactivity in EMA appears protocol-dependent. The coverage-based protocol was associated with modest reporting reactivity, which challenges the common assumption of reporting invariance in EMA and underscores the importance of considering protocol design when interpreting within-person change.

## Introduction

The rise of ecological momentary assessment (EMA) has transformed behavioral science by enabling the repeated sampling of behavior and experience in real time within natural environments [1,2]. By minimizing retrospective recall bias and capturing microprocesses as they unfold, EMA offers ecological validity and enhanced temporal resolution compared with traditional cross-sectional or laboratory-based methods. These advantages have made EMA an important tool for research on affect, stress, substance use, health behavior, and psychopathology [3-5]. However, the very feature that gives EMA its unique advantages (ie, intensive, repeated assessment) also raises a fundamental concern about potential reactive processes in self-report [6,7]: Does repeated self-report alter the reporting process itself? When individuals are repeatedly asked to monitor and report on their thoughts, emotions, or behaviors over a period of time, the assessment process itself may induce unintended reporting changes that may threaten the validity of the data. Repeated prompts may recalibrate internal standards, shift interpretations of what “counts,” reduce response thresholds through practice, or induce response fatigue [6,8-10]. If so, observed temporal trends in EMA data may reflect changes in reporting processes rather than genuine changes in the underlying construct.

This issue is closely tied to the concept of longitudinal measurement invariance [11]. In intensive longitudinal designs, where multiple observations per person are collected, measurement invariance across time is rarely tested [12]. For example, when self-reported stress declines across a week, it is typically interpreted as stress reduction. However, without an objective reference point, it is impossible to determine whether the underlying construct has changed or whether participants have altered the way they self-report. A growing body of research has demonstrated that repeated assessment may alter behavior itself (behavioral reactivity) across domains, including physical activity (PA), substance use, and affective processes [9,13-19].

In PA behaviors, a recent study showed a modest but consistent “initial burst” of activity among women with elevated cardiovascular disease risk, where they accumulated approximately 796 additional steps on the first day and 9.3 more minutes of moderate-to-vigorous PA during the first 2 days of a 10-day EMA protocol relative to the remainder of the study [20]. Similarly, another study found that older adults spent less time being sedentary and more time upright during the first 2 to 3 days of monitoring before returning toward habitual behavior [16]. Beyond behavioral outcomes, recent methodological work has shown that repeated assessment may alter the quality of self-report itself. A study analyzing data from 454 participants across 9 clinical EMA studies found that although response rates remained high (approximately 80%), careless responding increased and response variance decreased significantly over time, suggesting that repeated assessment may influence reporting processes even when compliance remains stable [21].

Collectively, these findings indicate that repeated assessment can influence behavior and response quality in different ways. However, comparatively little attention has been devoted to whether repeated assessment alters the reporting process independently of behavioral change (ie, reporting reactivity). This distinction is particularly important because most previous investigations of EMA reactivity have relied almost exclusively on self-report data, making it difficult to distinguish changes in the reporting process from genuine behavioral change. Consequently, longitudinal changes observed in EMA studies are typically interpreted as reflecting behavioral or psychological change under the assumption that reporting processes remain stable over time. Direct evaluation of this assumption requires the concurrent measurement of the underlying behavior using an external reference, an approach that has rarely been examined in EMA research.

In this study, we define self-report reactivity as a systematic, time-dependent change in the relationship between self-reported data and the underlying construct of interest. This may occur because repeated assessments may induce recalibration of internal standards, reconceptualization of constructs, practice effects, or response fatigue [22,23]. Such a shift in reporting processes is inherently developmental and can manifest as linear or nonlinear time trends in observed data.

Proper measurement of self-report reactivity requires an objective temporal anchor against which changes in self-report can be evaluated, a feature that has largely been absent from existing EMA research. PA and sedentary behavior (SB) provide a unique methodological opportunity because they can be measured concurrently using both EMA self-report and objective accelerometry. This concurrent measurement allows researchers to evaluate whether temporal changes in self-report correspond to changes in underlying behavior or instead reflect changes in the reporting process itself. Although accelerometry should not be considered a definitive measure of PA, it provides a good external behavioral reference against which temporal changes in self-report can be evaluated [24,25]. Stable differences between EMA and accelerometry are expected because the 2 methods capture related constructs. In contrast, systematic changes in their relationship over time are conceptually consistent with self-report reactivity and are therefore the focus of the present investigation.

In addition to testing for reporting reactivity, an important unanswered question is whether this divergence in temporal trajectories depends on features of the EMA protocol itself. Coverage-based protocols require participants to reconstruct behavior over extended recall windows (eg, the past 120 min), whereas near-momentary protocols focus on brief, immediate periods (eg, the past 5 min). Longer recall windows are hypothesized to increase cognitive reconstruction demands and anticipatory adjustments, potentially amplifying reporting reactivity. Despite these theoretical differences, there is limited empirical evidence regarding whether recall window length influences the longitudinal stability of self-reports. Comparing these 2 protocol designs, therefore, provides an opportunity to determine whether a modifiable feature of EMA methodology contributes to reporting reactivity.

Thus, the overall aim of this study was to evaluate whether repeated EMA is associated with reporting reactivity, operationalized as temporal divergence between EMA-reported and objectively measured behavior across repeated assessments. A secondary objective was to determine whether the magnitude of this divergence differed between coverage-based and near-momentary EMA protocols. To address these objectives, we evaluated whether temporal trajectories of EMA-reported PA and SB diverged from concurrently measured accelerometer-derived behavior across the monitoring period. Divergence between these trajectories would be consistent with reporting reactivity. We further examined whether the temporal pattern of this divergence differed between coverage-based and near-momentary protocols.

## Methods

### Study Design and Participants

This study conducted a secondary analysis of data collected from a larger study evaluating the validity of EMA-reported PA against objective accelerometry measures. The original study recruited participants from the Understanding America Study panel, a probability-based internet panel representing US residents [26], between January and July 2023. Recruitment was staggered throughout this period such that participants began the 7-day protocol on different days of the week, ensuring a balanced distribution of study start days across the calendar week to mitigate potential day-of-the-week confounds in behavioral patterns. Interested individuals were directed to an online screening survey to determine study eligibility. Eligibility criteria for the original study included being (1) at least 18 years old, (2) fluent in English, (3) having Wi-Fi access, and (4) having stable email access. Exclusion criteria were (1) existing hearing or visual impairment, (2) requiring an assistive mobility device, (3) working night shifts, or (4) being on bed rest. All participants provided informed consent. Upon enrollment, they were randomized to either a “coverage” EMA group (reporting on a 120-min window) or a “near-momentary” EMA group (reporting on a 5-min window). Study-provided Android smartphones (Motorola Moto G-Power 2021), preinstalled with the EMA application and waterproof accelerometers, were mailed to participants prior to study initiation. Participants completed an online demographic questionnaire and training modules before starting a 7-day EMA and accelerometry protocol. During the 7-day monitoring period, participants went through their usual daily activities while continuously wearing an accelerometer and answering EMA prompts (see details below). Participants returned both devices by mail following completion of the monitoring period and received financial compensation upon study completion. Data from both the coverage and near-momentary EMA groups were used in the current analysis to explore potential differences in reporting reactivity patterns.

### Ethical Considerations

All original study procedures were approved by the BRANY Institutional Review Board (study ID: #22-183-1044). All participants provided informed consent prior to enrollment. Participant data used for the present analyses were deidentified and stored on a secure drive to protect participant confidentiality. Participants received compensation of up to US $200, prorated based on their level of protocol completion.

### Measures

#### EMA

Over the 7-day study period, participants responded to EMA prompts delivered via the movisensXS app (version 1.8.2) installed on the study-provided smartphones. Five prompts were sent daily using a stratified random sampling design, occurring approximately every 3 to 4 hours during waking hours (6 AM to 10 PM), allowing for up to 35 assessments per individual. Participants also reported the sleep and wake times for the night during the first EMA prompt of each day. The EMA surveys queried participants about their PA and SB within the relevant time frame (120 min for the coverage group; 5 min for the near-momentary group). Participants were allowed to delay their responses to each EMA prompt for up to 15 minutes. After an initial alarm that lasted for 1 minute, reminder alarms lasting 1 minute each were administered by the app 5 and 10 minutes after the first alarm. The prompt expired 15 minutes after the beginning of the initial alarm, and the participant was unable to complete the EMA survey thereafter.

For this analysis, the key EMA variables were derived from participant responses regarding the duration of their activity. Specifically, participants reported the number of minutes spent in sedentary, standing, light, moderate, and vigorous activities within the specified windows (120 or 5 min; see Multimedia Appendix 1 for the list of relevant EMA questions). The light, moderate, and vigorous activities were summed to create a single measure of EMA-reported total active minutes. Participants also reported the time spent in sedentary activities, providing the EMA-reported sedentary minutes variable. While Verbal Rating Scale measures of activity and sedentariness were also collected, the analyses used the minute-based estimates. EMA items on affect, pain, and fatigue were also administered but were not analyzed here.

#### Accelerometry

Objective measurements of activity were obtained using a waterproof activPAL4 accelerometer (PAL Technologies Ltd), which participants wore continuously on the midline of their nondominant thigh for the entire 7-day period. The activPAL4 provides event-based classifications of posture and movement. Raw accelerometer data were processed using the activPAL software (PALbatch software, version 8.10.12.60) and the manufacturer’s GHLA classification algorithm (version 2.2). The GHLA algorithm classifies activity into sitting/lying, standing, and stepping events, from which posture and ambulatory behaviors were derived. Estimation of sleep periods involved a weighted-average algorithm that integrated self-reported sleep/wake times from EMA and activPAL-detected postural transitions; these sleep periods were excluded from analyses [27]. Periods identified as nonwear time by the activPAL were removed prior to analysis. EMA prompts were excluded from analyses when more than 90% of the corresponding accelerometer reporting window consisted of sleep or nonwear time, ensuring that self-reports aligned with objective measurements. For this study, accelerometer-derived sedentary time was operationalized as the total time classified as sitting or lying. Accelerometer-derived active behavior was operationalized as the total stepping time. Standing time was not included as active behavior because it does not represent ambulatory movement.

### Data Processing

To prepare the data for reactivity analyses, specific variables were derived from the processed EMA and accelerometry data. For each EMA prompt delivered to a participant, the accelerometry data corresponding to the relevant preceding time window—120 minutes for participants in the coverage group and 5 minutes for those in the near-momentary group—were extracted. For the EMA reports, instances where the self-reported EMA minutes exceeded the numeric threshold of the reporting window (eg, reporting >120 min within a 120-min window) were identified and replaced with the maximum possible value (which occurred in less than 3% of the data). Because the duration of the monitoring windows varied (120 min vs 5 min), raw minute counts were standardized to an hourly rate metric (minutes per hour). This metric was calculated by dividing the raw minutes of behavior by the window duration and multiplying by 60. This transformation placed both protocols on a common measurement scale. Each completed EMA prompt was paired with the corresponding time-matched accelerometry window immediately preceding the time at which the participant began responding to the EMA survey, rather than the time at which the prompt was initially delivered. The resulting prompt-level dataset preserved repeated within-person observations, with study day indicating the day of the monitoring period during which each matched observation occurred.

### Analytic Strategy

We used a multilevel modeling (MLM) strategy to assess reporting reactivity. This framework accounts for the hierarchical structure of the data, in which repeated prompt-level observations were nested within participants. Models included random intercepts and random slopes for both the linear and quadratic study day terms to account for individual differences in baseline behavior and temporal trajectories across the 7-day monitoring period. All models included day-of-the-week (eg, Monday, Tuesday) as a categorical covariate to control for typical weekly behavioral rhythms. All analyses were conducted in R (version 4.4.2) using the nlme package [28] for mixed-effects modeling. Estimated marginal means were obtained using the marginaleffects package [29] to visualize protocol-specific trajectories and their associated 95% CIs.

Because reporting reactivity is defined as a divergence in temporal trajectories between self-report and objective measurements, we used accelerometer-derived behavior within the EMA-matched time windows (ie, matching the time period being asked about in the EMA) as a temporal anchor. Objective behavior during these matched windows was examined to evaluate whether any trend was present across study days. Study day (coded 0‐6) was treated as a continuous predictor and modeled using linear and quadratic terms to detect potential temporal patterns.

Reporting reactivity was operationalized as the divergence in 7-day trajectories between EMA-reported behavior and time-matched accelerometer-derived behavior. In the absence of reporting reactivity, the trajectory of EMA-reported PA and SB should parallel that of objective behavior, even if absolute levels differ. To test this, we used a multivariable MLM approach. Data were structured in long format such that each completed EMA prompt contributed 2 outcome observations: one representing the EMA-reported behavior and the other representing the corresponding time-matched accelerometer-derived behavior. The model included dummy-coded variables for method (accelerometer=reference; EMA) and protocol (coverage=reference; momentary), linear and quadratic terms for study day (0=first day of monitoring), and all corresponding 2-way and 3-way interaction terms. The primary tests of reporting reactivity were the protocol-specific method × study day interactions, which evaluate whether the temporal trajectory of EMA-reported behavior diverged from that of the matched accelerometer measure *within* each protocol. Because coverage was the reference protocol, the method × study day coefficients directly represented EMA-accelerometer temporal divergence within the coverage protocol. For the near-momentary protocol, the corresponding effects were estimated as linear combinations of the method × study day and method × protocol × study day coefficients. Protocol-specific contrasts and their 95% CIs and *P* values were estimated using the variance-covariance matrix of the fixed effects. To determine whether reporting reactivity differed between protocols, method × protocol × study day interactions were examined. Significant 3-way interactions indicate that the magnitude or temporal pattern of EMA-accelerometer divergence differed between the coverage and momentary protocols.

Because the 5-minute and 120-minute protocols may differ in residual variability due to differences in temporal resolution, models were estimated using a heterogeneous residual variance structure (varIdent) that allowed separate residual variances for each protocol. This specification relaxes the assumption of homogeneous residual variance while retaining a unified modeling framework for protocol comparisons. Protocol-specific simple effects of the method × study day interaction were estimated using Wald tests of the corresponding contrasts.

## Results

### Participant Characteristics

Data for this analysis were drawn from participants in both the coverage and near-momentary EMA groups of the parent study. Overall, 315 respondents (148 in the coverage EMA group and 167 in the near-momentary EMA group) participated and completed the study, contributing 9917 prompts, yielding a 93% compliance rate (see Multimedia Appendix 2 for more details). After excluding prompts where over 90% of the associated reporting window consisted of accelerometer nonwear or sleep time, 3881 EMA observations with matched activPAL data remained for the coverage group and 4853 observations remained for the near-momentary group for the primary analyses. Participant demographic characteristics are summarized in Table 1. Briefly, participants were, on average, 49.2 (SD 14.9) years old, with a mean BMI of 29.7 (SD 7.9) kg/m^2^. Of the 315 participants, 47.6% (n=150) identified as male, 79.7% (n=251) identified as White, and 54.9% (n=173) had at least a bachelor’s degree.

**Table 1.** Demographic characteristics.

Characteristic	Overall (N=315)	Coverage (n=148)	Near-momentary (n=167)	*P* value
Age (y), mean (SD)	49.2 (14.9)	49.8 (14.9)	48.7 (15.0)	.50^a^
Gender, n (%)				.60^b^
Male	150 (47.6)	73 (49.3)	77 (46.1)	
Female	165 (52.4)	75 (50.7)	90 (53.9)	
Race, n (%)				.60^c^
White	251 (79.7)	121 (81.8)	130 (77.8)	
Black	22 (7.0)	12 (8.1)	10 (6.0)	
American Indian	4 (1.3)	2 (1.4)	2 (1.2)	
Asian	16 (5.1)	6 (4.1)	10 (6.0)	
Pacific Islander	2 (0.6)	0 (0)	2 (1.2)	
Prefer not to answer	20 (6.3)	7 (4.7)	13 (7.8)	
Education, n (%)				.60^b^
College educated	173 (54.9)	79 (53.4)	94 (56.3)	
Not college educated	142 (45.1)	69 (46.6)	73 (43.7)	
BMI (kg/m^2^), mean (SD)	29.7 (7.9)	30.0 (8.3)	29.3 (7.6)	.40^a^
Sedentary behavior (min/day), mean (SD)	633.7 (114.8)	639.1 (115.8)	628.9 (114.1)	.60^a^
Physical activity (min/day), mean (SD)	92.2 (41.7)	92.5 (43.1)	92.0 (40.6)	.9^a^2

a *P* values were calculated using the Wilcoxon rank-sum test.

b *P* values were calculated using the Pearson chi-square test.

c *P* values were calculated using Fisher exact test.

### Model-Estimated Behavioral Trajectories

Figure 1 presents model-estimated marginal means (Multimedia Appendix 3) derived from the final MLMs for EMA-reported and accelerometer-derived SB and PA across the 7-day monitoring period. Before assessing reactivity, a visual inspection of the lines (Figure 1; see Multimedia Appendix 3 for the marginal means) revealed a substantial main effect of measurement method: participants consistently reported different levels of SB and PA compared with accelerometry estimates. For instance, in the coverage group, the model-estimated objective SB during the time-matched window was 39.58 min/h, whereas EMA-reported SB was significantly lower at 33.12 min/h. A similar but more pronounced discrepancy was observed for PA, with objective estimates at 6.14 min/h vs self-reported estimates of 18.98 min/h. The near-momentary group showed similar patterns. Objective SB was estimated at 39.40 min/h compared with EMA-reported values of 35.83 min/h. Objective PA (5.71 min/h) was also substantially lower than EMA reports (25.26 min/h). While these discrepancies highlight a baseline difference between protocols, the primary goal of this study was to determine if reporting behavior changed systematically over the monitoring period. Thus, our analyses focused on the temporal trends (reactivity) rather than these overall differences.

**Figure 1.**
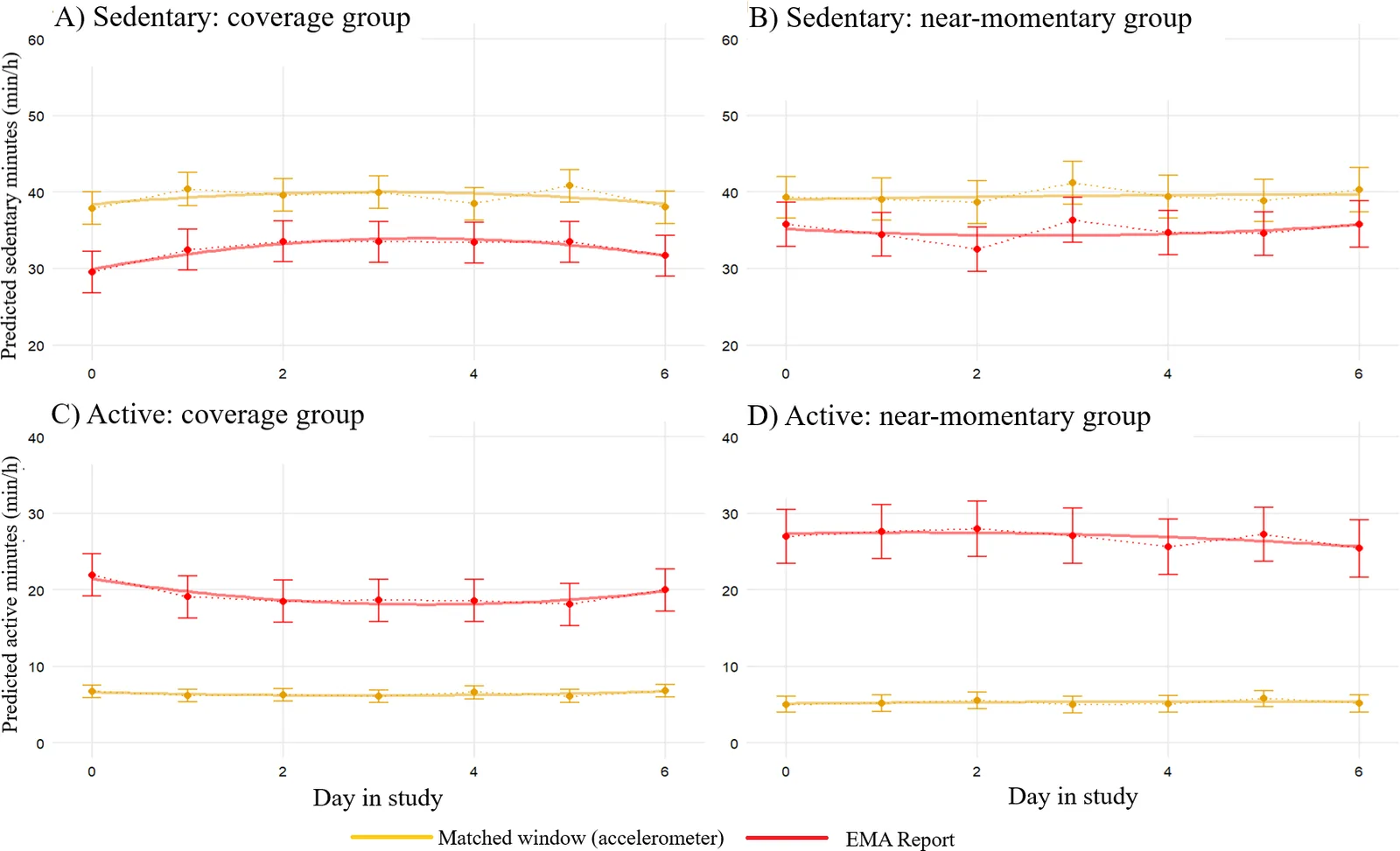
Trends in objective and self-reported sedentary and active behaviors across the 7-day monitoring period. Panels display modeled estimates for sedentary (A and B) and active (C and D) behaviors as hourly rates, stratified by the coverage group (left column) and momentary group (right column). Estimates are derived from the final multilevel model including the method × protocol × study day interaction. Solid semitransparent smooth lines represent the fitted polynomial trends. Solid dots represent the specific modeled marginal means, treating study day as a categorical factor, with vertical error bars indicating 95% CIs. EMA: ecological momentary assessment.

Across both protocols, accelerometer-derived behavior exhibited relatively stable trajectories, whereas temporal changes in EMA-reported behavior differed by protocol. Consistent with the observed trajectories, the coverage protocol demonstrated increasing divergence between EMA and accelerometer estimates across study days, whereas EMA and accelerometer trajectories were similar in the momentary protocol.

### Reporting Reactivity

We next formally tested the hypothesis that repeated monitoring was associated with reporting reactivity, operationalized as a divergence in the temporal trajectories between EMA-reported and accelerometry-based measurements. Model-estimated means and EMA-accelerometer contrasts were first examined to characterize the magnitude and pattern of these discrepancies within each protocol and study day (Figure 1; Multimedia Appendix 3). Protocol-specific contrasts of temporal trajectories were then used to formally test whether the EMA-accelerometer discrepancy changed systematically over the monitoring period (Table 2).

**Table 2.** Tests of protocol-specific temporal divergence between ecological momentary assessment (EMA) and accelerometer measurements from the final multilevel model including method × protocol × study day interactions.^a^

Hypothesis/contrast	Linear *b* (95% CI)	*P* value	Quadratic *b* (95% CI)	*P* value
Sedentary behavior				
Coverage protocol: temporal change in EMA-accelerometer discrepancy	1.40 (0.20 to 2.61)	.02	−0.19 (−0.38 to 0.01)	.06
Momentary protocol: temporal change in EMA-accelerometer discrepancy	−1.17 (−2.88 to 0.53)	.18	0.19 (−0.09 to 0.46)	.18
Difference in EMA-accelerometer temporal change between protocols	−2.58 (−4.66 to −0.49)	.02	0.38 (0.04 to 0.71)	.03
Physical activity				
Coverage protocol: temporal change in EMA-accelerometer discrepancy	−1.91 (−2.93 to −0.90)	<.001	0.28 (0.12 to 0.44)	<.001
Momentary protocol: temporal change in EMA-accelerometer discrepancy	−0.05 (−1.50 to 1.40)	.95	−0.04 (−0.27 to 0.20)	.74
Difference in EMA-accelerometer temporal change between protocols	1.86 (0.09 to 3.63)	.04	−0.32 (−0.60 to −0.03)	.03

a Values are unstandardized model-based estimates representing linear and quadratic changes in the EMA-accelerometer difference across study day. Temporal contrasts for the coverage protocol correspond to the method × study day interaction term in the model. For the near-momentary protocol, temporal contrasts were obtained as linear combinations of the method × study day and method × protocol × study day interaction terms. Corresponding 95% CIs and *P* values for these simple effects were calculated using Wald tests based on the estimated variance-covariance matrix of the fixed effects. Protocol comparison estimates correspond to the method × protocol × study day interaction.

In the coverage protocol, the model-estimated EMA-accelerometer discrepancy changed across the monitoring period. For SB, EMA underestimated accelerometer-derived sedentary time by 8.01 min/h on study day 0 (EMA-accelerometer contrast=−8.01, 95% CI −9.58 to −6.43 min/h). This discrepancy narrowed to 5.48 min/h on study day 3 (contrast=−5.48, 95% CI −6.48 to −4.48 min/h) before increasing to 6.29 min/h on study day 6 (contrast=−6.29, 95% CI −7.81 to −4.77 min/h). For PA, EMA overestimated accelerometer-derived activity by 15.16 min/h on study day 0 (contrast=15.16, 95% CI 13.83-16.48 min/h). The discrepancy narrowed to 11.91 min/h on study day 3 (contrast=11.91, 95% CI 11.07-12.75 min/h) before increasing to 13.64 min/h on study day 6 (contrast=13.64, 95% CI 12.35-14.92 min/h). Protocol-specific temporal contrasts formally tested whether these changes in the EMA-accelerometer discrepancy followed systematic linear or quadratic trends across study days. Because coverage was the reference protocol, the coverage-specific temporal contrasts corresponded directly to the method × study day interaction terms in the model. For SB, the linear component was significant (*b*=1.40, 95% CI 0.20-2.61, *P*=.02), whereas the quadratic component did not reach significance (*b*=−0.19, 95% CI −0.38 to 0.01, *P*=.06). For PA, both the linear (*b*=−1.91, 95% CI −2.93 to −0.90, *P*<.001) and quadratic (*b*=0.28, 95% CI 0.12-0.44, *P*<.001) components were significant, indicating that EMA-reported activity followed a curvilinear trajectory that diverged from the accelerometer trajectory.

In the near-momentary protocol, model-estimated EMA-accelerometer contrasts also demonstrated level differences between the 2 measurement methods, but these discrepancies showed less systematic change across study days. For SB, the estimated EMA-accelerometer contrast was −2.58 (95% CI −4.74 to −0.42) min/h on study day 0, reached −4.40 (95% CI −5.84 to −2.95) min/h on study day 3, and was −2.81 (95% CI −5.06 to −0.55) min/h on study day 6. For PA, the EMA-accelerometer contrast changed from 20.49 (95% CI 18.65-22.33) min/h on study day 0 to 19.99 (95% CI 18.75-21.22) min/h on study day 3 and 18.77 (95% CI 16.85-20.70) min/h on study day 6. The corresponding near-momentary protocol-specific temporal contrasts were estimated as linear combinations of the method × study day and method × protocol × study day terms. Neither the linear nor quadratic contrast reached statistical significance for SB (linear: *b*=−1.17, 95% CI −2.88 to 0.53, *P*=.18; quadratic: *b*=0.19, 95% CI −0.09 to 0.46, *P*=.18) or PA (linear: *b*=−0.05, 95% CI −1.50 to 1.40, *P*=.95; quadratic: *b*=−0.04, 95% CI −0.27 to 0.20, *P*=.74). Thus, we found no statistically detectable linear or quadratic change in the EMA-accelerometer discrepancy across study day in the near-momentary protocol.

### Coverage vs Near-Momentary Group Differences in Reporting Reactivity

Finally, we formally tested whether the trajectory of the EMA-accelerometer discrepancy across study day differed between protocols using the method × protocol × study day interactions. Significant 3-way interactions were observed for both SB (linear: *b*=−2.58, *P*=.02; quadratic: *b*=0.38, *P*=.03) and PA (linear: *b*=1.86, *P*=.04; quadratic: *b*=−0.32, *P*=.03), demonstrating that the temporal divergence between EMA and accelerometer measurements differed significantly between the coverage and momentary protocols (Multimedia Appendix 4). For example, for PA in the coverage-based protocol, the EMA-accelerometer discrepancy decreased from 15.16 min/h on day 0 to 11.91 min/h on day 3 before increasing to 13.64 min/h on day 6. In contrast, the discrepancy in the near-momentary protocol changed more gradually, from 20.49 min/h on day 0 to 19.99 min/h on day 3 and 18.77 min/h on day 6. A similar difference in temporal pattern was observed for SB. The coverage discrepancy changed from −8.01 min/h on day 0 to −5.48 min/h on day 3 and −6.29 min/h on day 6, whereas the near-momentary discrepancy changed from −2.58 to −4.40 and −2.81 min/h at the corresponding time points (Multimedia Appendix 3).

## Discussion

### Principal Findings

The central aim of this study was to evaluate whether repeated assessments in EMA were associated with reporting reactivity, defined as systematic temporal change in the discrepancy between EMA-reported and accelerometry-based measurements of the same behavior. Overall, the findings indicate that reporting reactivity was protocol-dependent. Participants assigned to the coverage protocol exhibited modest systematic divergence between EMA-reported and accelerometer-derived trajectories across the monitoring week, which was consistent with reporting-process change, whereas we found no evidence of such divergence in trajectories in the near-momentary protocol. Leveraging concurrent accelerometry as an external temporal anchor enabled direct evaluation of stability of reporting process, which is a common assumption in EMA research but is rarely examined empirically because objective reference measures are typically unavailable.

In the coverage protocol, the EMA-accelerometer discrepancy changed systematically across the monitoring period for both PA and SB. These changes followed a curvilinear pattern, with the discrepancy narrowing during the early-to-middle portion of the monitoring period and partially rebounding thereafter. In contrast, we found no statistically detectable linear or quadratic temporal change in the EMA-accelerometer discrepancy in the near-momentary protocol. Importantly, the significant method × protocol × study day interactions indicated that the shape of these discrepancy trajectories differed between protocols, rather than indicating that temporal divergence occurred generally across both protocols. The initial pattern mimics much of the existing reactivity literature, which describes an “initial burst” or elevation effect where self-report is heightened in the first 1 or 2 days of monitoring [6].

This phenomenon can be understood through several mechanisms. As discussed, participants may have recalibrated their internal scale for judging the duration or intensity of their activity (*beta change*) or redefined what activities “count” toward their reports (*gamma change*) as a result of repeated measurement [22]. The EMA prompts may also have compelled participants to create new cognitions or beliefs about their behavior simply to answer the questions posed [23], further altering their reports. The initial overreporting of desirable behavior (PA) or underreporting of undesirable behavior (SB) on day 1 in our study was also suggestive of a social desirability effect, which then appeared to dissipate over the week as participants engaged in these other reactive processes. The partial rebound observed later in the monitoring period may reflect the emergence of additional processes, including survey fatigue or changes in participants’ engagement with repeated assessments. Because these mechanisms were not measured directly, they should be regarded as plausible explanations rather than definitive interpretations. Future studies should incorporate measures of response fatigue, perceived burden, and reporting recalibration to better distinguish among these potential mechanisms.

These findings extend previous work on EMA reactivity. Much of the existing literature has focused on whether repeated assessment changes behavior itself, affects compliance, or alters overall levels of self-reported responses [6,13,14,16,18]. In contrast, the present study evaluated whether repeated assessment changes the reporting process by examining temporal divergence between self-report and concurrent objective measurements. This distinction is important because reporting reactivity may occur even in the absence of behavioral reactivity, yet both phenomena can produce similar longitudinal patterns in EMA data. Importantly, systematic temporal change in the discrepancy between EMA and accelerometry, as observed in the coverage protocol, presents a challenge for EMA research. In a longitudinal intervention study, such a shift could be dangerous for inferences about change over time. A natural improvement in reporting accuracy over time could be mistaken for a decline in PA, potentially masking a true treatment effect. Consequently, failing to model this nonstationary error may introduce significant confounds, particularly in studies examining dynamic processes.

Consistent with prior literature, we observed substantial level differences between EMA-reported and accelerometer-measured behavior. EMA overestimated PA behavior and underestimated SB relative to objective measurements. These discrepancies are not unexpected. Self-report measures are influenced by recall error, cognitive heuristics, social desirability, and differences in construct definitions (eg, what participants consider “activity” vs what an accelerometer detects). However, our goal was not to evaluate the absolute validity of EMA relative to accelerometry. Level discrepancies alone may not pose a threat, especially in EMA studies where within-person processes are often the main focus of interest. In this context, even imperfect self-report measures can support valid longitudinal inference as long as reporting bias is stable. It is the nonstationarity of reporting error that poses the greater threat to internal validity.

These findings may have implications for EMA methodology beyond the domain of PA outcomes. In domains such as affect, stress, or pain, where objective anchors are unavailable, researchers assume longitudinal measurement invariance. Our results suggest that this assumption may not always hold, particularly when extended recall windows are used. Reporting reactivity may pose as behavioral change, intervention effects, or symptom improvement. If similar reporting drift occurs in these domains, apparent symptom improvement or behavioral change could partially reflect changes in reporting processes rather than changes in the underlying construct.

Apart from reporting reactivity, another contribution of this study is that it examined behavioral reactivity in a setting where the participants are being overtly and actively monitored. Accelerometry alone is often described as passive monitoring, but when paired with repeated EMA prompts that explicitly ask participants to reflect on their recent behavior, the salience of being monitored is heightened. If behavioral reactivity were to occur, it would plausibly emerge under these conditions. However, we observed little evidence of systematic change in objectively measured behavior across the study period. This suggests that even when monitoring is both passively and actively reinforced through repeated reporting, behavioral reactivity in daily PA may be limited in magnitude. Of course, the lack of a counterfactual means that we cannot rule out the possibility that a reactive effect may manifest as a level change in actual behavior that is stable across the entire study period (eg, participants decided to exercise 30 min every day throughout the study period because they were being monitored).

### Strengths and Limitations

This study has several notable strengths. A major strength of this study is that we leveraged concurrent accelerometry as an objective anchor, allowing us to evaluate whether the trajectories of EMA-reported behavior diverged from the objective measure. In most EMA research, such divergence cannot be assessed because no objective anchor exists. By using PA behavior as a methodological vehicle, we were able to examine an important assumption in most EMA studies regarding the stability of reporting processes across time. Second, the randomized protocol comparison enabled us to isolate the impact of recall window length on reporting reactivity. Given the proliferation of coverage-like protocols in EMA studies, our findings underscore the importance of considering how this specific design choice might introduce reporting drift when interpreting within-person change.

The study also has several limitations that warrant consideration. First, the monitoring window was 1 week; longer monitoring periods may reveal different developmental patterns. Second, PA represents a specific, observable behavioral domain. Self-report reactivity may manifest differently in more abstract constructs such as affect and other internal states. It should also be noted that, while concurrent accelerometry serves as an objective temporal anchor, it is not a perfect measure of PA behaviors [25]. Furthermore, our analyses focused on temporal trends across the monitoring week rather than shorter within-day fluctuations in reporting processes, potentially masking acute, within-day survey fatigue. Additionally, while we standardized reporting to an hourly rate, differences in protocol discrepancy (coverage vs momentary) may partially reflect the scale and variance structure of reporting error inherent in 120-minute vs 5-minute recall windows rather than differential reactivity alone. To account for this, we employed a heterogeneous residual variance structure in our models, though structural differences in cognitive recall demands cannot be entirely eliminated. Finally, the absence of a no-monitoring control group limits causal inference regarding behavioral reactivity. To characterize these effects, future research should move beyond secondary analyses and use prospective experimental designs. Specifically, studies that systematically manipulate the active ingredients of EMA study protocols (eg, salience of reporting expectancies or the presence of performance feedback) are critical to isolating the specific mechanisms that drive measurement reactivity. As EMA increasingly serves both assessment and intervention functions, understanding when and how monitoring alters behavior and reporting becomes not merely a methodological concern, but a foundational question for ambulatory science.

### Conclusion

In conclusion, this study provides some evidence consistent with reporting reactivity in EMA, but this was contingent on the EMA design. Using concurrent accelerometry as a temporal anchor, we found systematic temporal change in the EMA-accelerometer discrepancy within the coverage protocol, whereas no statistically detectable linear or quadratic temporal change was observed in the near-momentary protocol. The temporal pattern of this discrepancy also differed significantly between protocols. These findings suggest that the assumption of longitudinal stability in self-report—an important premise in most EMA studies—may not always hold. Future research should experimentally manipulate EMA design features to identify the mechanisms that drive self-report reactivity and to strengthen the psychometric foundations of intensive longitudinal methods.

## Supplementary material

10.2196/99953 Multimedia Appendix 1 Relevant ecological momentary assessment questions.

10.2196/99953 Multimedia Appendix 2 Ecological momentary assessment compliance across the monitoring period.

10.2196/99953 Multimedia Appendix 3 Model-estimated marginal means (95% CIs) for sedentary behavior and physical activity by study day, protocol, and measurement method.

10.2196/99953 Multimedia Appendix 4 Fixed-effect estimates from the multilevel models evaluating temporal divergence between ecological momentary assessment and accelerometer measurements.

## Abbreviations

EMA: ecological momentary assessment
MLM: multilevel modeling
PA: physical activity
SB: sedentary behavior
