# Supplementary material for: Temporal Stability of Repeated Self-Reported vs Device-Measured Behavior: Observational Study Using Ecological Momentary Assessment
Source: J Med Internet Res. 2026 Oct 23;28:e99953. doi: 10.2196/99953 (PMC13717333; doi:10.2196/99953)
Supplement: Supplementary file 1 [file jmir-v28-e99953-s001.docx]

Multimedia Appendix I

Relevant EMA Questions

| **Behavior** | **Question wording** | **Response type** |
| --- | --- | --- |
| **Sedentary** | During the [5/120] minutes before the prompt, how many minutes did you spend sitting or lying down? (e.g., you were awake in a seated posture and not expending enough effort to raise your heart rate) | (Numeric value input) “minute(s)” |
| **Standing** | During the [5/120] minutes before the prompt, how many minutes did you spend standing? Please only include time spent standing still. | (Numeric value input) “minute(s)” |
| **Light** | During the [5/120] minutes before the prompt, how many minutes did you spend in light activities? Light activities refer to activities that take minimal physical effort and cause little to no elevation in your heart rate such as walking, light yard work or housework like washing dishes or cooking. | (Numeric value input) “minute(s)” |
| **Moderate** | During the [5/120] minutes before the prompt, how many minutes did you spend in moderate activities? Moderate activities refer to activities that take moderate physical effort and make you breathe somewhat harder than normal. Examples of moderate physical activities include, but are not limited to, bicycling at a regular pace, or playing double tennis. | (Numeric value input) “minute(s)” |
| **Vigorous** | During the [5/120] minutes before the prompt, how many minutes did you spend in vigorous activities? Vigorous activities refer to activities that take hard physical effort and make you breathe much harder than normal. Examples of vigorous physical activities include, but are not limited to, heavy lifting, digging, aerobics, or fast bicycling. | (Numeric value input) “minute(s)” |
