# Supplementary material for: Temporal Stability of Repeated Self-Reported vs Device-Measured Behavior: Observational Study Using Ecological Momentary Assessment
Source: J Med Internet Res. 2026 Oct 23;28:e99953. doi: 10.2196/99953 (PMC13717333; doi:10.2196/99953)
Supplement: Supplementary file 2 [file jmir-v28-e99953-s002.docx]

Multimedia Appendix 2

EMA Compliance Across the Monitoring Period

To evaluate whether differential EMA compliance across the monitoring period could provide an alternative explanation for the protocol-specific temporal patterns observed in the primary analyses, we examined all EMA prompts delivered to participants included in the analytic sample prior to accelerometer matching and other prompt-level exclusions. Compliance was defined as the proportion of delivered EMA prompts that were completed. Overall compliance was similar between protocols participants. In the Coverage protocol completed 4,683 of 5,004 delivered prompts (93.6%), whereas participants in the near-Momentary protocol completed 5,234 of 5,637 prompts (92.9%). Completion rates also remained consistently high across the seven-day monitoring period, ranging from 92.3% to 94.8% in the Coverage protocol and from 91.4% to 94.3% in the near-Momentary protocol (Supplementary Table S3).

Supplemental Table S3. EMA compliance rates by study day and protocol

| **Study day** | **Coverage, n/N (%)** | **Near-Momentary, n/N (%)** |
| --- | --- | --- |
| 0 | 660/715 (92.3%) | 767/827 (92.7%) |
| 1 | 673/710 (94.8%) | 759/820 (92.6%) |
| 2 | 675/720 (93.8%) | 763/816 (93.5%) |
| 3 | 673/711 (94.7%) | 762/808 (94.3%) |
| 4 | 680/722 (94.2%) | 739/797 (92.7%) |
| 5 | 660/713 (92.6%) | 734/792 (92.7%) |
| 6 | 662/713 (92.8%) | 710/777 (91.4%) |
| **Overall** | **4683/5004 (93.6%)** | **5234/5637 (92.9%)** |

To formally evaluate whether compliance changed differently across the monitoring period between protocols, we fit a mixed-effects logistic regression predicting prompt completion from protocol, Study Day (coded 0–6), and their interaction, with a participant-specific random intercept. There was no evidence of an overall linear change in completion across Study Day in the Coverage protocol (*b* = −0.004, SE = 0.065, *p* = .954), nor was there evidence that the temporal pattern of completion differed between the Coverage and near-Momentary protocols (Protocol × Study Day: *b* = −0.010, SE = 0.040, *p* = .799). These findings indicate that EMA completion remained relatively stable across the monitoring week in both protocols and provide little evidence that differential compliance over time accounted for the protocol-dependent patterns observed in the primary analyses.
