# Supplementary material for: Temporal Stability of Repeated Self-Reported vs Device-Measured Behavior: Observational Study Using Ecological Momentary Assessment
Source: J Med Internet Res. 2026 Oct 23;28:e99953. doi: 10.2196/99953 (PMC13717333; doi:10.2196/99953)
Supplement: Supplementary file 3 [file jmir-v28-e99953-s003.docx]

| Model-estimated marginal means (95% confidence intervals) for sedentary behavior and physical activity by study day, protocol, and measurement method. | | | | | | | | |
| --- | --- | --- | --- | --- | --- | --- | --- | --- |
|  |  | **Sedentary behavior (mins/hour)** | | |  | **Physical Activity (mins/hour)** | | |
| **Study Day** | | **ACC estimate  (95% CI)** | **EMA Estimate  (95% CI)** | **EMA − ACC contrast  (95% CI)** |  | **ACC Estimate  (95% CI)** | **EMA Estimate  (95% CI)** | **EMA − ACC contrast (95% CI)** |
| **Coverage** | | |  |  |  |  |  |  |
|  | 0 | 38.62 (36.76, 40.48) | 30.61 (28.75, 32.47) | −8.01 (−9.58, −6.43) |  | 6.33 (4.81, 7.84) | 21.49 (19.96, 23.01) | 15.16 (13.83, 16.48) |
|  | 1 | 39.53 (38.12, 40.95) | 32.74 (31.33, 34.15) | −6.79 (−7.75, −5.84 |  | 6.05 (4.90, 7.20) | 19.57 (18.42, 20.73) | 13.52 (12.72, 14.32) |
|  | 2 | 40.10 (38.69, 41.51) | 34.15 (32.74, 35.56) | −5.95 (−6.88, −5.02) |  | 5.88 (4.76, 6.99) | 18.32 (17.20, 19.44) | 12.44 (11.66, 13.22) |
|  | 3 | 40.31 (38.85, 41.77) | 34.83 (33.37, 36.30) | −5.48 (−6.48, −4.48) |  | 5.81 (4.65, 6.96) | 17.72 (16.56, 18.88) | 11.91 (11.07, 12.75) |
|  | 4 | 40.17 (38.75, 41.59) | 34.80 (33.37, 36.22) | −5.38 (−6.31, −4.44) |  | 5.84 (4.68, 6.99) | 17.77 (16.61, 18.93) | 11.93 (11.15, 12.72) |
|  | 5 | 39.68 (38.23, 41.13) | 34.04 (32.58, 35.49) | −5.65 (−6.59, −4.71) |  | 5.97 (4.77, 7.18) | 18.48 (17.27, 19.69) | 12.51 (11.72, 13.30) |
|  | 6 | 38.84 (36.90, 40.78) | 32.55 (30.61, 34.49) | −6.29 (−7.81, −4.77) |  | 6.21 (4.66, 7.75) | 19.85 (18.29, 21.40) | 13.64 (12.35, 14.92) |
| **Momentary** | | |  |  |  |  |  |  |
|  | 0 | 38.78 (36.71, 40.85) | 36.20 (34.12, 38.27) | −2.58 (−4.74, −0.42) |  | 5.47 (3.75, 7.19) | 25.97 (24.24, 27.69) | 20.49 (18.65, 22.33) |
|  | 1 | 39.24 (37.74, 40.73) | 35.67 (34.18, 37.17) | −3.56 (−4.89, −2.24) |  | 5.50 (4.27, 6.74) | 25.91 (24.66, 27.15) | 20.40 (19.27, 21.53) |
|  | 2 | 39.56 (38.06, 41.07) | 35.39 (33.88, 36.90) | −4.17 (−5.51, −2.83) |  | 5.53 (4.31, 6.75) | 25.76 (24.54, 26.98) | 20.23 (19.09, 21.37) |
|  | 3 | 39.75 (38.17, 41.33) | 35.36 (33.78, 36.94) | −4.40 (−5.84, −2.95) |  | 5.55 (4.27, 6.82) | 25.53 (24.25, 26.81) | 19.99 (18.75, 21.22) |
|  | 4 | 39.81 (38.29, 41.33) | 35.56 (34.04, 37.08) | −4.25 (−5.58, −2.91) |  | 5.56 (4.31, 6.81) | 25.22 (23.97, 26.47) | 19.66 (18.52, 20.80) |
|  | 5 | 39.73 (38.17, 41.29) | 36.01 (34.45, 37.57) | −3.71 (−5.08, −2.35) |  | 5.57 (4.26, 6.87) | 24.82 (23.52, 26.13) | 19.25 (18.09, 20.42) |
|  | 6 | 39.51 (37.30, 41.73) | 36.71 (34.49, 38.92) | −2.81 (−5.06, −0.55) |  | 5.57 (3.76, 7.38) | 24.34 (22.53, 26.16) | 18.77 (16.85, 20.70) |
| Values represent model-estimated marginal means (total minutes per hour) with 95% confidence intervals derived from the final multilevel model. Estimates are averaged across the day-of-week covariate and correspond to the fitted trajectories displayed in Figure 1. EMA − ACC contrasts represent model-estimated contrasts between EMA-reported and accelerometer-derived behavior within each protocol and study day, with 95% confidence intervals calculated for the contrast. | | | | | | | | |

Multimedia Appendix 3
