# Supplementary material for: Temporal Stability of Repeated Self-Reported vs Device-Measured Behavior: Observational Study Using Ecological Momentary Assessment
Source: J Med Internet Res. 2026 Oct 23;28:e99953. doi: 10.2196/99953 (PMC13717333; doi:10.2196/99953)
Supplement: Supplementary file 4 [file jmir-v28-e99953-s004.docx]

Multimedia Appendix 4

| Fixed-effect estimates from the multilevel models evaluating temporal divergence between EMA and accelerometer measurements. | | | | | | | |
| --- | --- | --- | --- | --- | --- | --- | --- |
| **Predictor** | **SB *b*** | **SE** | **p-value** |  | **PA *b*** | **SE** | **p-value** |
| **Intercept** | 38.62 | 0.95 | <.001 |  | 6.33 | 0.77 | <.001 |
| **Method (EMA vs ACC [ref])** | -8.01 | 0.8 | <.001 |  | 15.16 | 0.68 | <.001 |
| **Protocol (Momentary vs Coverage [ref])** | 0.16 | 1.42 | 0.912 |  | -0.85 | 1.17 | 0.466 |
| **Study Day (Linear)** | 1.09 | 0.61 | 0.073 |  | -0.33 | 0.45 | 0.469 |
| **Study Day (Quadratic)** | -0.18 | 0.1 | 0.074 |  | 0.05 | 0.07 | 0.47 |
| **Day of Week (Monday)** | -1.04 | 0.48 | 0.029 |  | 0.49 | 0.38 | 0.195 |
| **Day of Week (Tuesday)** | 1.03 | 0.51 | 0.045 |  | -0.42 | 0.4 | 0.29 |
| **Day of Week (Wednesday)** | -0.95 | 0.43 | 0.026 |  | 0.84 | 0.35 | 0.016 |
| **Day of Week (Thursday)** | -0.13 | 0.39 | 0.746 |  | -0.15 | 0.33 | 0.646 |
| **Day of Week (Friday)** | 0.1 | 0.38 | 0.802 |  | 0.05 | 0.32 | 0.886 |
| **Day of Week (Saturday)** | -0.37 | 0.38 | 0.33 |  | 0.34 | 0.32 | 0.288 |
| **Method × Protocol** | 5.43 | 1.36 | <.001 |  | 5.33 | 1.16 | <.001 |
| **Method × Study Day (Linear)** | 1.4 | 0.61 | 0.022 |  | -1.91 | 0.52 | <.001 |
| **Method × Study Day (Quadratic)** | -0.19 | 0.1 | 0.056 |  | 0.28 | 0.08 | <.001 |
| **Protocol × Study Day (Linear)** | -0.56 | 0.95 | 0.557 |  | 0.36 | 0.74 | 0.625 |
| **Protocol × Study Day (Quadratic)** | 0.11 | 0.15 | 0.486 |  | -0.05 | 0.12 | 0.645 |
| **Method × Protocol × Study Day (Linear)** | -2.58 | 1.06 | 0.015 |  | 1.86 | 0.9 | 0.04 |
| **Method × Protocol × Study Day (Quadratic)** | 0.38 | 0.17 | 0.028 |  | -0.32 | 0.15 | 0.03 |
| SB = Sedentary behavior. PA = Physical activity. EMA = Ecological momentary assessment. ACC = Accelerometer. Beta values are unstandardized fixed-effect parameter estimates from the final multilevel model. Method compares EMA with accelerometer measurements, Protocol compares the Momentary and Coverage EMA protocols, and Study Day was modeled using linear and quadratic terms. Day of Week was modeled as an orthogonal polynomial with linear through sixth-order components. Random intercepts and random slopes for Study Day (linear and quadratic) were included for participants, and protocol-specific residual variances were estimated using a heterogeneous variance structure. | | | | | | | |
